# Sex Differences in Baseline Characteristics Do Not Predict Early Outcomes after Percutaneous Coronary Intervention: Results from the Australian GenesisCare Cardiovascular Outcomes Registry (GCOR)

**DOI:** 10.3390/jcm11041138

**Published:** 2022-02-21

**Authors:** Andre Conradie, Sinny Delacroix, MyNgan Duong, Nisha Schwarz, Enayet Chowdhury, Stephen Worthley, John Atherton, David Eccleston

**Affiliations:** 1 Cardiology Department, Friendly Society Private Hospital, 23 Bingera Street, Bundaberg, QLD 4670, Australia; 2 Cardiology Department, GenesisCare, Buildings 1&11, The Mill, 41–43 Bourke Road, Alexandria, NSW 2015, Australia; sinny.delacroix@genesiscare.com (S.D.); my-ngan.duong@genesiscare.com (M.D.); nisha.schwarz@genesiscare.com (N.S.); enayet.chowdhury@genesiscare.com (E.C.); stephen.worthley@genesiscare.com (S.W.); 3 Cardiology Department, Royal Brisbane and Women’s Hospital, Butterfield St., Herston, QLD 4029, Australia; john.atherton@health.qld.gov.au; 4 Cardiology Department, Melbourne Private Hospital, Royal Parade, Parkville, VIC 3052, Australia; david.eccleston@genesiscare.com

**Keywords:** acute coronary syndrome, PCI, sex

## Abstract

Objective: The effect of baseline differences between men and women on early outcomes after percutaneous coronary intervention (PCI). Design, setting, participants: This is an observational study of all participants in the GenesisCare Cardiovascular Outcomes Registry, undergoing PCI. The registry holds data for both emergency and elective procedures. Data was collected on 10,989 consecutive patients from 12 Australian Private Hospitals, including baseline demographics, co-morbidities, risk factors, PCI procedures, and lesion characteristics. Main outcome measures: Outcome was measured for complications (in-hospital death, peri-procedural myocardial infarctions, and bleeding events), at discharge and at 30-days for death, myocardial infarction, target lesion revascularisation (TLR), major adverse cardiac events (MACE), and unplanned readmissions. Results: Women represented 23% of the study population, were significantly older, with a higher rate of hypertension and hyperlipidaemia. Heart failure was more common in women and was associated with a significantly higher average ejection fraction than in men. Women had a lower rate of pre-existing coronary artery disease (CAD), had less complex CAD, and needed fewer stents. Periprocedural complications were similar, but major bleeding was more common in women. The 30-day outcome was similar between men and women for death, myocardial infarction, target lesion revascularisation (TLR), major adverse cardiovascular events (MACE), and unplanned readmissions. Conclusions: Although significant differences were observed between women and men in both clinical presentation and complexity of disease, the 30-day outcome was similar for death and MACE. Women had a higher rate of major bleeding events, and lower adherence to statins and dual antiplatelet therapy (DAPT).

## 1. Introduction

Cardiovascular disease (CVD) is one of the leading causes of death in women worldwide including USA and Australia [1,2]. Most of the studies on outcome and increased mortality in women undergoing percutaneous intervention (PCI) has focussed on acute coronary syndromes (ACS), with information on chronic stable angina mostly derived from registries and sub-studies [1].

In patients, presenting with ACS, studies have reported women to suffer increased morbidity and a higher risk of mortality following an index myocardial infarction (MI) event [2,3,4,5]. This has often been attributed to delayed diagnosis, and decreased access to angiography and reperfusion resulting from sex-based disparities in the clinical presentation [6]. Women more commonly present with non-ST elevation MI (NSTEMI) and a higher probability of non-obstructive CAD or normal coronary arteries at angiography [6]. Non-atherosclerotic mechanisms could partly explain this with women having a higher rate of spontaneous coronary artery dissection (SCAD), Takotsubo syndrome, and coronary artery spasm [3,4,7]. From a symptom perspective, both men and women reported similar rates of chest pain, but a higher rate of atypical symptoms has been noted for women, especially in <55-year-olds, delaying diagnosis and lifesaving therapies including reperfusion therapy and percutaneous intervention (PCI) [6]. Another possible contributing factor to a lower diagnostic rate of ACS in women compared to men, is the lower levels of cardiac troponin and creatine kinase-myocardial band (CK-MB) in women. In a study by Shah et al. [8], having different diagnostic thresholds of high sensitivity Troponin I (TnI) (16 ng/L for women vs. 34 ng/L for men) rather than a single threshold of 50 ng/L, had a two-fold increase in the diagnosis of MI in women. Furthermore, some studies have also reported that women presenting with ACS younger than 55-years of age have an added higher out-of-hospital cardiac arrest, progression to heart failure and mortality [3,4,5].

On the contrary, data on the outcome for chronic stable angina patients presenting for PCI (i.e., undergoing elective PCI) have mostly been reported from large registries which included an ACS cohort as well as patients with chronic stable angina. These include the British Cardiovascular Intervention Society (BCIS) and the Swedish Coronary Angiography and Angioplasty Registry (SCAAR) registries [9,10] which have identified female sex as an independent predictor of 30-day mortality.

Definite biological and sex-based differences have been identified that potentially contribute to the difference in outcome and increased mortality in women. Women present at an older age with CAD and with a higher burden of co-morbidities [6,7,11]. A very low rate of premature CAD (<55-years) has also been identified in women which can partially be explained by the protective role of oestrogen [4]. Additionally, studies such as the INTERHEART study have also reported that the majority of risk factors for CAD are common to both men and women [12], except certain factors such as diabetes, smoking, and psychosocial factors which account for a greater risk for CAD in women compared to men. Women were noted to have a higher burden of these risk factors and often presented with ≥3 risk factors at the index MI event.

Although there are definite sex-based differences in women and men presenting with CAD and treated with PCI, the correlation of these sex-based differences to in-hospital and 30-day mortality in an Australian cohort is unclear. Further research is warranted to not only address and delineate the underlying disparities, but also to further understand the higher mortality risk seen in women, especially in a high-risk population undergoing PCI in a real-world scenario. Additionally, to our knowledge the current literature on early outcomes in women and men following PCI largely focuses on the ≥12-month timepoint. This study is well placed wherein a large PCI registry, with a combination of acute (undergoing emergency PCI) and patients with stable CAD (undergoing elective PCI and at high-risk) can be assessed for sex-based differences. This study aims to analyse demographic, clinical, and procedural characteristics including 30-day outcomes for both sexes, utilizing data from patients undergoing PCI in a large private clinical cohort in Australia.

## 2. Method

### 2.1. Study Setting and Participants

The GenesisCare Cardiovascular Outcomes Registry (GCOR-PCI), established in 2008, is an internally funded ongoing prospective registry for patients undergoing PCI at 12 Australian private hospitals. The analysis for this study includes data from patients enrolled between January 2009 and December 2017. Details of the GCOR registry were published earlier [13]. Briefly, GCOR data parameters and definitions were developed using interventional databases including the American College of Cardiology (ACC) and the National Cardiovascular Data Registry (NCDR^®^) guidelines. Data are entered consecutively for procedures using a web-based electronic case report form (eCRF). Clinical management and interventions are determined by the treating cardiologist using published guidelines on medical therapy.

Validity of available data is checked routinely by GenesisCare’s data management team in quarterly audits. At each audit, 5% of all individual records are randomly extracted from each site (which can total ≥500 records) and compared to source data for ~50 randomly selected data-fields. The 2019 audit reports revealed high data quality with ~98% accuracy in data collection. The systems and processes of data collection and records comply with the privacy and data protection guidelines relevant to Health Records and Information Privacy Acts and Information Privacy Principles. This study is included in the Australian New Zealand Clinical Trials Registry (ANZCTR number ACTRN12620000899943).

### 2.2. Baseline Measures, Follow-Up, and Clinical Outcomes

Socio-demographic parameters, medical history and management including in-hospital investigations and procedure details are routinely recorded. All in-hospital complications following the procedure are recorded at the time of discharge. Follow-up was performed by research coordinators at discharge and at 30-days post-procedure. All cardiac events are documented following the review of medical records including death, myocardial infarction (MI), target lesion revascularisation (TLR, defined as revascularisation within 5 mm of a previously treated segment), and target vessel revascularisation (TVR, defined as revascularisation of a previously treated artery). Major adverse cardiovascular events (MACE), defined as the composite of death, MI, and/or TVR, were also collected.

### 2.3. Statistical Analyses

Descriptive analyses included frequencies, sample proportions or mean with standard deviations to summarise baseline patient characteristics (e.g., age, clinical parameters, etc.), risk factors, and procedural characteristics. Student’s *t*-test, ANOVA or chi-square tests were used to compare the distributions of baseline characteristics including procedural and/or risk factors by sex. Further, the association of clinical outcomes and medication use at discharge and 30-day follow-up was compared between men and women using univariate and multivariate logistic regression. Multivariate logistic regression models were adjusted for patient age, baseline clinical characteristics (i.e., diabetes, hypertension, BMI, previous history CVD, PCI) and clinical presentation (i.e., ST-elevation MI (STEMI)/NSTEMI, multivessel disease). Adjusted variables were the known risk factors for sex-based differences for CAD [1,12,14]. All statistical analyses were performed using Stata version 14.1 for Windows (StataCorp LP, College Station, TX, USA).

## 3. Results

### 3.1. Demographics and Risk Profile at Index Presentation

A total of 10,989 consecutive patients from the GCOR-PCI database were included in the study. Detailed risk profiles are presented in Table 1. The distribution of CAD by race was uneven with 96.5% being Caucasian and 3.5% from other ethnicities. Women represented 23% of the study population, with the average age of women being significantly older than men (71.6 ± 10.3 years for women vs. 67.3 ± 10.5 years for men; *p* < 0.001). When assessing the mean age at the time of clinical presentation, women presented more commonly at an older age (>75 years) compared to men, and less commonly at a younger age (<55 years) (*p* < 0.001). The distribution of risk factors was very similar across both men and women with the exception of hypertension (80.2% for women vs. 71.9% for men; *p* < 0.001) and hyperlipidaemia (88.8% for women vs. 85.2% for men, *p* < 0.001) which was higher in women. Differences in smoking status between men and women was statistically significant with fewer women being current smokers (6.8% for women vs. 9.3% for men; *p* < 0.001), or with a previous history of smoking (61.7% for men vs. 39.7% for women; *p* < 0.001). Women were less likely to present with a history of previous CAD, with a lower risk of previous MI (19.5% for women vs. 24.2% for men, *p* < 0.001), previous PCI (28.8% for women vs. 33.6% for men, *p* < 0.001), or previous coronary artery bypass grafting (CABG) (8.7% for women vs. 12.3% for men, *p* < 0.001). No significant difference was noted by sex for the presence of peripheral vascular disease (PVD) (*p* = 0.071), or renal impairment (*p* = 0.583). Women were more likely to present with heart failure (5.1% in women vs. 2.9% in men; *p* < 0.001), and more likely to have a previous history of heart failure (5.9% in women vs. 4.6% in men, *p* = 0.009). In patients presenting with ACS (23%), the risk of Killip class III to IV was higher in women compared to men (*p* = 0.015), and the risk for cardiogenic shock was significantly higher in women (0.7% for women vs. 0.3% for men; *p* = 0.008). The need for intra-aortic balloon pump (IABP) support, when presenting with ACS, was significantly higher in women compared to men (0.8% in women vs. 0.3% in men; *p* = 0.002). The average ejection fraction at presentation for a PCI was higher in women compared to men (56.7 ± 10.1 for men vs. 58.1 ± 10.4 for women, *p* < 0.001). Similar distribution was noted for STEMI for both sexes, however, women had a higher risk of presenting with NSTEMI (*p* = 0.042) and unstable angina (*p* = 0.023) (Table 1).

### 3.2. Procedure and Lesion Characteristics

Statistically significant differences were observed by sex for both femoral and radial access (Table 2). Femoral access was more commonly utilised in women compared to men (71.9% for women vs. 67.9% for men, *p* < 0.001), with radial access being significantly lower in women compared to men (27.8% in women vs. 31.7% in men, *p* < 0.001). The proportion of patients with de novo lesions was similar for both sexes and there was a non-significant trend with women more likely to present with in-stent restenosis (6.1% in women vs. 5.2% in men, *p* = 0.087). Complete total occlusion of the index lesion was less common in women (3.5% in women vs. 4.7% in men, *p* = 0.016). When complexity of the lesions was assessed by ACC/AHA guidelines a statistically significant difference was observed by sex. Women presented more often with type A lesions (14.9% in women vs. 12.5% in men; *p* = 0.003) and, less often with more complex lesions (46.8% in women vs. 51.0% in men, *p* < 0.001). When target vessels were assessed, a significant higher proportion of women presented with left anterior descending artery (LAD) lesions compared to men (45.8% in women vs. 41.5% in men; *p* < 0.001). No difference was noted for bifurcation lesions (*p* = 0.86), and procedural success was similar for both sexes (97.0% for men vs. 96.8% for women, *p* = 0.60) (Table 2).

Fractional Flow Reserve (FFR) measurement was more often utilised in women compared to men (12.3% in women vs. 9.5% in men, *p* < 0.001). The use of thrombus aspiration devices (2.2% in men vs. 2.0% in women) and rotablators (2.3% in men vs. 2.7% in women) were very low and similar in both sexes (Table 2).

Women were more likely to receive bare metal stents (BMS) (13.7% in women vs. 12.0% in men, *p* = 0.026) and less likely to receive a drug eluting stent (DES) (78.3% in women vs. 80.5% in men, *p* = 0.024). Women were less likely to have multivessel disease (37.4% in women vs. 44.6% in men, *p* < 0.001). Pathology in smaller vessels (≤2.5 mm) was more likely in women compared to men (32.9% in women vs. 23.4% in men, *p* < 0.001), and this was coupled with significantly lower average stent diameters (2.9 ± 0.4 mm in women vs. 3.0 ± 0.5 mm in men, *p* < 0.001) and stent lengths (17.9 ± 5.9 in women vs. 18.8 ± 6.2 mm in men, *p* < 0.001) in women compared to men. The average number of stents used per procedure were also less in women compared to men (1.3 in women vs. 1.4 in men, *p* < 0.001). No difference in the use of P_2_Y_12_ receptor blockers were noted, with most patients receiving a loading dose during the procedure (Table 2).

### 3.3. Clinical Events and Outcomes at Discharge and 30-Days

The risk of in-hospital mortality was higher in women compared to men (0.3% in men vs. 0.6% in women, *p* = 0.014); however, this was no longer significant when adjusted for baseline variables (OR 1.31; 95% CI 0.40 to 4.24, *p* = 0.66). The rate of peri-procedural myocardial infarction was similar for both men and women (2.2% in men vs. 2.3% in women, *p* = 0.806). Women had a statistically significant increase in bleeding events at discharge (3.4% in women vs. 1.7% in men, *p* < 0.001), with women experiencing a significantly higher rate of BARC (Bleeding Academic Research Consortium) 3–5 bleeding (1.2% in women vs. 0.2% in men, *p* < 0.001). At 30-day follow-up, no difference was noted for rate of death, MI, TLR, MACE and unplanned readmissions (Table 3).

### 3.4. Medication Use at Baseline and Adherence at 30 Days

The use of the P_2_Y_12_-receptor blockers was significantly lower in women compared to men (OR 0.74; 95% CI 0.60 to 0.90, *p* = 0.003) unlike aspirin use, which was similar across both sexes at discharge (OR 0.99; 95% CI 0.68 to 1.45, *p* = 0.96). Although not clinically significant, these trends persisted at 30-day follow-up. Women were more likely to be prescribed beta-blockers at discharge. On the other hand, women were less likely to be prescribed statins at discharge and 30-day follow-up (*p* < 0.001 for both timepoints). No difference was seen between the groups for the use of angiotensin-converting enzyme inhibitors (ACEI) or angiotensin receptor blockers (ARB) at both time points (Table 4).

## 4. Discussion

This study confirms differences in clinical characteristics and procedural outcomes between women and men presenting with ACS and undergoing PCI. Significant differences were observed not only in the clinical presentation, but also in the complexity of disease with a higher risk of peri-procedural bleeding in women following PCI. Additionally, women were less likely to receive DAPT or statins and more likely to receive beta blockers. Interestingly, women had a higher percentage of in-hospital deaths compared to men, however, the female sex was not associated with an increased risk of mortality in adjusted analyses.

Previous studies assessing patients with ACS undergoing PCI (ACC-NCDR), demonstrated that women presenting with CAD are not only older, but also have more comorbidities [15]. The GCOR database revealed a higher prevalence of hypertension and hyperlipidaemia as risk factors in women (*p* < 0.001), with a much smaller percentage of women being current smokers (*p* < 0.001). Notably, the risk of diabetes was similar for men and women, while body mass index (BMI) was significantly lower in women in our study compared to the study from ACC-NCDR [15]. These differences observed might be related to selection bias or possibly a reflection of the socio-economic bias observed for patients in private facilities.

This study observed a significantly higher proportion of women presenting with symptoms of heart failure (including historical) but with high left ventricular ejection fraction (*p* < 0.001) and a higher prevalence of hypertension in women suggestive of diastolic dysfunction seen in patients with heart failure with preserved ejection fraction (HFpEF). Very similar to the ACC-NCDR database [15], women in the GCOR database had a lower incidence of previous CAD (MI, PCI, or CABG), but demonstrated a higher prevalence of previous cerebro-vascular disease (CeVD). The possible explanations for the higher incidence of previous CeVD could be the higher rate of hypertension and hyperlipidaemia noted in this group which was coupled with a trend of more women having atrial fibrillation (15.4% of women vs. 13.7% of men, *p* = 0.103). Lower adherence to secondary prevention measures in women might also be a contributing factor, with significantly fewer women adherent to statins.

From observational studies and contemporary databases (ACC-NCDR), women presented more often with unstable angina or NSTEMI compared to men (82% women vs. 77% men) and had a higher risk of cardiogenic shock [9,15]. The results presented in this study confirm the same trend, with 23% women vs. 21.1% men presenting with NSTEMI, and 18.8% women vs. 16.8% men presenting with unstable angina. In the GCOR database women presented with a higher Killip score, and cardiogenic shock was present in 0.7% of women vs. 0.3% of men (*p* = 0.008). Factors that might explain this, include an older age group contributing to more complex CAD, and a higher presence of pre-existing heart failure and hypertension in women.

The GCOR database confirmed a significantly higher bleeding risk in women compared to men (3.4% in women vs. 1.7% in men, *p* < 0.001). This was in line with most trials and registries, including the GRACE registry [16], where female sex was identified as an independent risk predictor for major bleeding, even after adjusting for baseline differences, including age, weight, and renal function [10,12,17]. A possible contributing factor in this study for the higher bleeding risk observed in women is the much higher rate of femoral access and lower rate of radial procedures (*p* < 0.001) in women. In the GRACE registry, the most common cause of major bleeding in patients undergoing PCI was vascular access site bleeding, while the overall increased risk of bleeding during hospitalisation was 43% higher for women compared to men [16]. The difference in radial and femoral approaches, however, was not unique to this study, with similar findings noted from the BCIS and SCAAR registries [9]. The higher rate of femoral access may be explained by women having smaller vessels and a higher risk of radial artery spasm, often resulting in crossover rate from radial to femoral access (7% in the SAFE-PCI trial) [4].

In patients undergoing PCI, the female sex has been associated with a higher risk of mortality and MACE compared to men. Both the BCIS and SCAAR registries confirmed the female sex to be an independent predictor of 30-day mortality using multiple regression analysis [9]. In the GCOR database, the risk of overall in-hospital mortality was very low (0.4%), albeit, with a higher rate noted in women compared to men (0.6 vs. 0.3%, *p* = 0.014). On the other hand, similar rates of peri-procedural MI were observed in both sexes (*p* = 0.806). At 30-days no sex-based differences were noted for both all-cause mortality (*p* = 0.861) and MACE (*p* = 0.090). A possible explanation for this current finding is that only a small percentage of the GCOR cohort presented with STEMI and the majority of these patients underwent an elective procedure. This possible effect on outcome was assessed by performing a sub-analysis comparing patients presenting with ACS with elective PCI (Supplementary Tables S1 and S2), however, no significant difference was demonstrated. An additional contributing factor could be the lower rate of co-morbidities in the GCOR cohort compared to other registries. That said, these findings were in line with the ACC-NCDR registry, that reported an adjusted odds ratio for in-hospital mortality similar for both men and women (OR 0.97, *p* = 0.5) [15].

Secondary prevention measures are important to prevent complications post-PCI, such as in-stent thrombosis, restenosis, cardiovascular death, and MACE. Evidence based medicine has resulted in a significant decline in cardiovascular mortality in women over the last 10 years [4]. The SWEDEHEART registry noted a significant reduction in re-infarction, stroke, and heart failure that was directly related to the increased use of antiplatelet therapy, betablockers, ACE/ARB inhibition, and statins. This resulted not only in a short-term benefit, but also in improved 1-year outcomes [18]. Despite this evidence of benefit, women were less likely to receive aspirin or statin therapy at discharge [15]. This was also evident in the GCOR database with fewer women receiving aspirin on discharge (96.8% in women vs. 97.8% in men, *p* = 0.006), and an even more significant difference observed in the treatment with P_2_Y_12_ receptor inhibitors (90.3% in women vs. 93.2% in men, *p* < 0.001). This difference in antiplatelet prescriptions did not affect peri-procedural MIs, and the numbers of in-hospital mortality were too small to determine a clinical effect. At 30-days, a similar reduced rate of aspirin and P_2_Y_12_-receptor inhibitor use was noted in women, with no difference in mortality or MACE by sex. A similar trend was seen for compliance with statins, with the use of statins being significantly lower in women both at discharge as well as at 30-days follow-up (91.6% in women vs. 94.2% in men, *p* < 0.001). Medication adherence in the GCOR data set for both aspirin and P_2_Y_12_ inhibitors at discharge revealed the same trend for lower use in women, but a significantly lower rate of P_2_Y_12_ use was seen in the GCOR registry compared to the ACC-NCDR registry (96.8 vs. 94.7% for aspirin, 90.3 vs. 95.0% for clopidogrel). A similar difference was seen with statins, with a lower rate of use in women, however, a significantly larger percentage of women were discharged on statins compared to the ACC-NCDR registry (91.3 vs. 81.0% for statins), suggestive of lack of medication adherence or compliance measures potentially from both patients and physicians [15].

### Limitations

This study provides real-world evidence on patients from various geographic locations in Australia presenting with CAD for a PCI. However, limitations were noted with this study. This study included only patients undergoing PCI and therefore was not representative of all patients with CAD. Another possible limiting factor was the very low event rate, especially for mortality, despite the overall large study size. Including patients mostly from private centres might also have influenced the outcome, as well as the lower rate of patients presenting with ACS. The study reported only on the 30-day outcome, with the possibility that more significant differences could be demonstrated over a longer follow-up period.

## 5. Conclusions

This study confirmed that women presenting at an older age with CAD, had a lower rate of pre-existing CAD, and were more clinically indisposed at presentation with a higher rate of heart failure and need of hemodynamic support. Women had less complex CAD at presentation and a higher average left ventricular ejection fraction. A significantly higher major bleeding rate was noted in women, with the unadjusted in-hospital mortality higher in women. Despite significant differences between women and men at presentation, no difference in mortality and MACE was noted at 30-days. Further research is needed to investigate the underlying reasons for the higher unadjusted in-hospital mortality in women as well as the lower adherence to antiplatelet therapy and statins. The association of heart failure in women with higher average left ventricular ejection fraction suggests that diastolic function is more prevalent in women, and the possible impact on long-term prognosis in women requires further ongoing investigation.

## Figures and Tables

**Table 1:** Demographics and risk profile.

	Overall	Men	Women	*p*-Value
Number of procedures	10,989	8479 (77%)	2507 (23%)	
Mean age in years	68.2 ± 10.6	67.3 ± 10.5	71.6 ± 10.3	<0.001
<55 years	11.5	12.8	7.2	<0.001
55–74 years	60.8	63.1	53.3	
>75 years	27.7	24.1	39.5	
Race				
Caucasian	96.5	96.3	97.4	0.022
Aboriginal/Torres Strait	0.1	0.1	0.1	
Other	3.4	3.6	2.5	
Risk Factors				
Diabetes	24.3	24.1	25.1	0.350
Hypertension	73.8	71.9	80.2	<0.001
Hypercholesterolaemia	86.0	85.2	88.8	<0.001
Family History of CAD	40.5	40.1	42.2	0.063
Previous history of Heart failure	4.9	4.6	5.9	0.009
Current heart failure	3.4	2.9	5.1	<0.001
Smoking				
Never smoked	44.7	39.7	61.7	<0.001
Previous smoker	46.6	51.0	31.5	
Current smoker	8.7	9.3	6.8	
BMI	28.97 ± 5.24	29.07 ± 4.87	28.64 ± 6.30	<0.001
Previous MI	23.2	24.2	19.5	<0.001
Previous PCI	32.5	33.6	28.8	<0.001
PVD	7.4	7.1	8.2	0.071
CVD	7.1	6.8	8.4	0.006
CABG	11.5	12.3	8.7	<0.001
Renal Impairment %	5.3	5.4	5.1	0.583
>90		24.4	20.0	<0.001
60–89		55.1	50.7	<0.001
45–59		13.2	17.0	<0.001
30–44		5.3	8.4	<0.001
15–29		1.2	3.0	<0.001
<15		0.8	0.8	0.985
Atrial fibrillation (*n* = 6412)	14.1	13.7	15.4	0.103
**Clinical Presentation**:				
Killip class: (*n* = 2517)				
I	87.9	88.7	85.5	0.015
II	8.6	8.5	9.0	
III	2.0	1.7	2.8	
IV	1.5	1.2	2.7	
Ejection fraction% (mean ± SD)	57.1 ± 10.2	56.7 ± 10.1	58.1 ± 10.4	<0.001
<40%		5.2	4.7	0.355
40–49%		9.4	7.4	0.003
>50%		85.4	87.9	0.003
IABP	0.4	0.3	0.8	0.002
Cardiogenic shock	0.4	0.3	0.7	0.008
PCI Presentation				
Elective	53.9	54.7	51.2	0.002
STEMI	7.3	7.4	7.0	0.501
NSTEMI	21.5	21.1	23.0	0.042
Unstable angina	17.3	16.8	18.8	0.023

CAD—Coronary artery disease; BMI—Body mass index; PVD—Peripheral vascular disease; CVD—Cerebrovascular disease; CABG—Coronary artery bypass grafting; IABP—Intra-aortic balloon pump therapy; STEMI—ST-elevation myocardial infarction; NSTEMI—Non-ST-elevated myocardial infarction; SD—Standard deviation.

**Table 2:** Procedure and lesion characteristics.

	All (*n* = 10,986)		
	Men	Women	*p*
Number of procedures	8479	2507	NA
Number of lesions	11,750	3304	NA
Lesions per procedure, mean ± SD	1.4 ± 0.63	1.3 ± 0.58	<0.001
Multi-vessel disease among procedures	44.6	37.4	<0.001
Access Site			
Femoral access	67.9	71.9	<0.001
Radial Access	31.7	27.8	<0.001
Other/Brachial	0.4	0.3	0.422
Type of Lesions			
De Novo	93.6	93.2	0.58
Restenosis	0.4	0.2	0.064
In-stent Restenosis	5.2	6.1	0.087
Other	0.8	0.5	0.117
CTO/Total occlusion	4.7	3.5	0.016
ACC/AHA Morphology			
A	12.5	14.9	0.003
B_1_	36.4	38.3	0.112
B_2_.C	51.0	46.8	<0.001
Target Vessel			
RCA	31.1	32.6	0.17
LMCA	1.5	1.2	0.29
LAD	41.5	45.8	<0.001
LCX	21.7	17.5	<0.001
GRAFT/Bypass	4.2	2.9	0.005
Bifurcation Lesion (Provisional Stenting)	9.8	10.0	0.86
Intracoronary Device			
FFR any	9.5	12.3	<0.001
Thrombus aspiration device any	2.2	2.0	0.49
Rotablator any	2.3	2.7	0.35
BMS any	12.0	13.7	0.026
DES any	80.5	78.3	0.024
Stents (among lesions):			
Total number	11,701	3158	NA
Average stents per procedure; mean ± SD	1.4 ± 0.9	1.3 ±0.9	<0.001
Stent length (mm); mean ± SD	18.8 ± 6.2	17.9 ± 5.9	<0.001
Stent length > 20 mm; (%, among all stent)	30.9	26.1	<0.001
Stent diameter (mm); mean ± SD	3.0 ± 0.5	2.9 ± 0.4	<0.001
Vessel ≤ 2.5 mm	23.4	32.9	<0.001
Procedural Success	97.0	96.8	0.60
Procedural Complications			
Acute closure	0.6	0.8	0.25
Dissection	2.5	2.8	0.50
Perforation	0.3	0.2	0.64
No reflow	2.9	3.1	0.73

RCA—Right coronary artery; LMCA—Left main coronary artery; LAD—Left anterior descending artery; LCX—Left circumflex artery; FFR—Fractional flow reserve; BMS—Bare metal stent; DES—Drug—eluting stent; SD—Standard deviation.

**Table 3:** Clinical events and outcomes at discharge and 30-days.

	Men	Women	*p* Value (Unadjusted)	Adjusted Odds Ratio * (95% Confidence Interval) in Women (vs. Men)	*p* Value
Number of Procedures	8479	2507	NA	-	-
**Discharge**					
Discharged	8455	2500	NA		
Death	25 (0.3)	15 (0.6)	0.014	1.31 (0.40–4.24)	0.66
Myocardial Infarction	186 (2.2)	57 (2.3)	0.806	0.89 (0.62–1.28)	0.55
Bleeding events	140 (1.7)	85 (3.4)	<0.001	2.02 (1.38–2.96)	<0.001
**30-Day Follow up**					
Eligible Procedures	8441	2489	NA		
Followed up *n* (%)	8304 (98.4)	2444 (98.2)	0.526		
Death *n* (%)	15 (0.18)	4 (0.16)	0.861	0.57 (0.11–2.92)	0.50
MI *n* (%)	12 (0.14)	3 (0.12)	0.800	1.31 (0.30–5.72)	0.72
TVR *n* (%)	34 (0.41)	3 (0.12)	0.033	0.55 (0.16–1.94)	0.36
TLR *n* (%)	21 (0.25)	4 (0.16)	0.421	0.56 (0.12–2.60)	0.46
MACE *n* (%)	59 (0.71)	10 (0.41)	0.105	0.81 (0.36–1.82)	0.61
Unplanned Readmission *n* (%)	193 (2.32)	64 (2.62)	0.402	1.18 (0.84–1.67)	0.34

* Adjusted for age, diabetes, hypertension, hypercholesteremia, BMI, heart failure, smoking status, previous history of CVD, PCI/CABG, eGFR, LVEF, PCI presentation and multivessel disease (yes/no). MI—myocardial infarction; TVR—Target vessel revascularization; TLR—Target lesion revascularization; MACE—Major adverse cardiovascular events.

**Table 4:** Medication use at discharge and 30 days.

	Men %	Women %	*p*-Value (Unadjusted)	Adjusted Odds Ratio * (95% Confidence Interval) in Women (vs. Men)	*p*-Value (Adjusted)
**Drug Therapy**					
***During Discharge***	*n* = 8479	*n* = 2507			
Aspirin	97.8	96.8	0.006	0.99 (0.68–1.45)	0.96
Clopidogrel/Prasugrel/Ticagrelor	93.2	90.3	<0.001	0.74 (0.60–0.90)	0.003
Statin	94.2	91.3	<0.001	0.67 (0.53–0.85)	0.001
B-Blocker	58.1	58.7	0.563	1.14 (1.01–1.28)	0.04
ACE/ARB	69.2	67.3	0.067	0.92 (0.80–1.05)	0.21
Anti-arrhythmic	3.9	5.8	<0.001	1.11 (0.84–1.45)	0.47
***At 30 Day Follow-up***	*n* = 8304	*n* = 2444			
Aspirin	96.1	95.1	0.034	0.97 (0.72–1.30)	0.84
Clopidogrel/Prasugrel/Ticagrelor	90.2	88.8	0.053	0.89 (0.73–1.07)	0.22
Statin	94.2	91.6	<0.001	0.65 (0.52–0.83)	<0.001
B-Blocker	55.2	56.2	0.365	1.11 (0.98–1.25)	0.11
ACE/ARB	67.4	66.2	0.281	0.93 (0.82–1.06)	0.27
Anti-arrhythmic	3.3	4.2	0.061	0.93 (0.67–1.31)	0.69

* Adjusted for age, diabetes, hypertension, hypercholesteremia, BMI, heart failure, smoking status, previous history of CVD, PCI/CABG, eGFR, lvef, pci presentation, and multivessel disease (yes/no). ACE—Angiotensin-converting enzyme inhibitors; ARB—Angiotensin II receptor blockers.

## Data Availability

The datasets generated during and/or analysed during the current study are available from the corresponding author on reasonable request.

## Supplementary Materials

The following are available online at https://www.mdpi.com/article/10.3390/jcm11041138/s1, Table S1: Outcomes in patients undergoing elective and emergency PCI; Table S2: Medications in patients undergoing elective and emergency PCI.
