# Supplementary material for: Sex Differences in Baseline Characteristics Do Not Predict Early Outcomes after Percutaneous Coronary Intervention: Results from the Australian GenesisCare Cardiovascular Outcomes Registry (GCOR)
Source: J Clin Med. 2022 Feb 21;11(4):1138. doi: 10.3390/jcm11041138 (PMC8877078; doi:10.3390/jcm11041138)
Supplement: Supplementary file 1 [file jcm-11-01138-s001.zip › jcm-1567690-supplementary.pdf]

Supplementary

Table S1. Outcomes in patients undergoing elective and emergency PCI.

	Elective							Urgent/Emergency						
	**Men**	**Women**	***p*-value**	**Odds ratio***	**95% conf. Interval**		**Adj. *p*-value**	**Men**	**Women**	***p*-value**	**Odds ratio**	**95% conf. interval**		**Adj. *p*-value**
Discharged	4564	1265		-	-	-	-	3779	1210	-	-	-	-	-
Death	1 (<1%)	0 (0.0%)	0.60	-				21 (0.6%)	15 (1.2%)	0.014	1.56	0.47	5.21	0.467
Myocardial Infarction	92 (2.0%)	27 (2.2%)	0.81	0.95	0.57	1.59	0.855	92 (2.5%)	30 (2.5%)	0.91	0.82	0.49	1.38	0.456
Bleeding events	68 (1.5%)	32 (2.5%)	0.012	1.66	0.92	3.02	0.095	67 (1.8%)	52 (4.3%)	<0.001	2.33	1.41	3.86	0.001
Followed up n (%)	4516	1253		-	-	-	-	3694	1170		-	-	-	-
Death n (%)	0 (0.00%)	2 (0.16%)	0.007	-	-	-	-	15 (0.41%)	2 (0.17%)	0.230	0.29	0.03	2.47	0.257
MI n (%)	7 (0.16%)	0 (0.00%)	0.160	-	-	-	-	5 (0.14%)	3 (0.26%)	0.370	3.77	0.65	21.98	0.140
TVR n (%)	16 (0.35%)	3 (0.24%)	0.530	1.21	0.30	4.83	0.789	16 (0.43%)	0 (0.00%)	0.024	-	-	-	-
TLR n (%)	11 (0.24%)	2 (0.16%)	0.580	-	-	-	-	10 (0.27%)	2 (0.17%)	0.550	3.77	0.65	21.98	0.140
MACE n (%)	22 (0.49%)	5 (0.40%)	0.690	1.23	0.37	4.07	0.735	35 (0.95%)	5 (0.43%)	0.086	0.58	0.19	1.77	0.338
Unplanned Readmission n (%)	96 (2.13%)	23 (1.84%)	0.520	0.97	0.57	1.63	0.898	95 (2.57%)	41 (3.50%)	0.092	1.47	0.92	2.36	0.105

* Odds ratio and corresponding *p*-value are adjusted for age, diabetes, hypertension, hypercholesteremia, BMI, heart failure, smoking status, previous history of CVD, PCI, CABG, eGFR, LVEF, and presence of multivessel disease.

Table S2. Medications in patients undergoing elective and emergency PCI.

	Elective							Urgent/Emergency						
	**Men**	**Women**	***p*-value**	**Odds ratio***	**95% conf. interval**		**Adj. *p*-value**	**Men**	**Women**	***p*-value**	**Odds ratio**	**95% conf. interval**		**Adj. *p*-value**
Discharge	4563	1265	-	-	-	-	-	3759	1195	-	-	-	-	-
Aspirin	4453 (97.9%)	1223 (96.8%)	0.019	0.76	0.46	1.25	0.281	3684 (98.1%)	1164 (97.7%)	0.36	1.40	0.78	2.52	0.266
Clopidogrel/Prasugrel/ Ticagrelor	4178 (91.6%)	1100 (87.0%)	<0.001	0.66	0.52	0.84	0.001	3600 (95.8%)	1136 (95.1%)	0.30	0.93	0.65	1.33	0.697
Statin	4243 (93.6%)	1142 (90.3%)	<0.001	0.61	0.45	0.83	0.002	3575 (95.5%)	1108 (93.3%)	0.003	0.78	0.54	1.14	0.198
B-Blocker	2320 (51.1%)	661 (52.3%)	0.45	1.21	1.03	1.43	0.023	2515 (67.0%)	789 (66.2%)	0.62	1.04	0.87	1.23	0.698
ACE/ARB	3072 (67.4%)	817 (64.6%)	0.063	0.86	0.72	1.04	0.123	2710 (72.1%)	849 (71.0%)	0.48	0.97	0.80	1.17	0.731
Anti-arrhythmic	174 (3.8%)	70 (5.5%)	0.008	1.04	0.70	1.55	0.855	148 (3.9%)	70 (5.9%)	0.005	1.21	0.83	1.75	0.324
30 Day follow-up	4516	1253		-	-	-	-	3694	1170		-	-	-	-
Aspirin	4168 (96.3%)	1154 (95.1%)	0.041	0.84	0.56	1.24	0.376	3362 (95.8%)	1042 (95.2%)	0.38	1.17	0.75	1.83	0.477
Clopidogrel/Prasugrel/ Ticagrelor	3944 (88.9%)	1050 (84.9%)	<0.001	0.67	0.54	0.85	0.001	3316 (92.0%)	1045 (93.1%)	0.22	1.46	1.04	2.06	0.030
Statin	4045 (93.7%)	1095 (90.5%)	<0.001	0.55	0.41	0.75	0.000	3322 (94.8%)	1011 (92.8%)	0.009	0.82	0.57	1.18	0.290
B-Blocker	2111 (48.9%)	605 (49.8%)	0.57	1.13	0.96	1.34	0.145	2202 (62.9%)	690 (63.2%)	0.85	1.05	0.88	1.25	0.626
ACE/ARB	2913 (65.7%)	781 (63.2%)	0.1	0.87	0.72	1.05	0.140	2498 (69.4%)	777 (69.3%)	0.95	0.98	0.81	1.18	0.825
Anti-arrhythmic	137 (3.3%)	43 (3.7%)	0.52	0.80	0.49	1.31	0.385	106 (3.3%)	44 (4.5%)	0.075	1.08	0.68	1.71	0.749

* Odds ratio and corresponding *p*-value are adjusted for age, diabetes, hypertension, hypercholesteremia, BMI, heart failure, smoking status, previous history of CVD, PCI, CABG, eGFR, LVEF, and presence of multivessel disease.
